# MicroRNA and Cardiovascular Diseases

**DOI:** 10.4274/balkanmedj.galenos.2020.2020.1.94

**Published:** 2020-02-28

**Authors:** Hüseyin Altuğ Çakmak, Mehmet Demir

**Affiliations:** 1 Clinic of Cardiology, Mustafakemalpaşa State Hospital, Bursa, Turkey; 2 Department of Cardiology, University of Health Sciences, Bursa Yüksek İhtisas Research and Training Hospital, Bursa, Turkey

**Keywords:** Cardiovascular disease, gene expression, microRNAs

## Abstract

Cardiovascular diseases are one of the most common causes of death in both developing and developed countries worldwide. Even though there have been improvements in primary prevention, the prevalence of cardiovascular diseases continues to increase in recent years. Hence, it is crucial to both investigate the molecular pathophysiology of cardiovascular diseases in-depth and find novel biomarkers regarding the early and proper prevention and diagnosis of these diseases. MicroRNAs, or miRNAs, are endogenous, conserved, single-stranded non-coding RNAs of 21-25 nucleotides in length. miRNAs have important roles in various cellular events such as embryogenesis, proliferation, vasculogenesis, apoptosis, cell growth, differentiation, and tumorigenesis. They also have potential roles in the cardiovascular system, including angiogenesis, cardiac cell contractility, control of lipid metabolism, plaque formation, the arrangement of cardiac rhythm, and cardiac cell growth. Circulating miRNAs are promising novel biomarkers for purposes of the diagnosis and prognosis of cardiovascular diseases. Cell or tissue specificity, stability in serum or plasma, resistance to degradative factors such as freeze-thaw cycles or enzymes in the blood, and fast-release kinetics, provide the potential for miRNAs to be surrogate markers for the early and accurate diagnosis of disease and for predicting middle- or long-term prognosis. Moreover, it may be a logical approach to combine miRNAs with traditional biomarkers to improve risk stratification and long-term prognosis. In addition to their efficacy in both diagnosis and prognosis, miRNA-based therapeutics may be beneficial for treating cardiovascular diseases using novel platforms and computational tools and in combination with traditional methods of analysis. microRNAs are promising, novel therapeutic agents, which can affect multiple genes using different signaling pathways. miRNAs therapeutic modulation techniques have been used in the settings of atherosclerosis, acute myocardial infarction, restenosis, vascular remodeling, arrhythmias, hypertrophy and fibrosis, angiogenesis and cardiogenesis, aortic aneurysm, pulmonary hypertension, and ischemic injury. This review presents detailed information about miRNAs regarding structure and biogenesis, stages of synthesis and functions, expression profiles in serum/plasma of living organisms, diagnostic and prognostic potential as novel biomarkers, and therapeutic applications in various diseases.

Cardiovascular diseases (CVD) are one of the most common causes of death in both developing and developed countries worldwide (1). Even though there have been improvements in primary prevention, the prevalence of CVD continues to increase in recent years. Hence, it is crucial to both investigate the molecular pathophysiology of CVD in-depth and find novel biomarkers regarding the early and proper prevention and diagnosis of these diseases. While nearly 80% of genes in the human body undergo transcription, only 1% to 2% of them get translated into proteins, which leaves many non-coding RNA (ncRNA) transcripts (2,3). ncRNAs are composed of small nuclear and nucleolar RNAs, PIWI-interacting RNAs, Y-RNAs, microRNAs (miRNAs), and long ncRNAs. ncRNAs are very important in regulating gene expression and for using epigenetic applications. Furthermore, they may be one of the most important etiologic factors for the development of CVD. To date, miRNAs are the most studied and characterized ncRNAs in the literature (4).

miRNAs are endogenous, conserved, single-stranded non-coding RNAs of 21-25 nucleotides in length (5). Lin-4, which is the first miRNA, was discovered in Caenorhabditis elegans in 1993. Moreover, the primary miRNA database was released in 2002 with only 218 entries, and they continued to increase over the following years. The latest miRBase Sequence Database includes 28,645 entries standing for hairpin precursor miRNAs, which includes 35,828 mature miRNAs in 223 species. After that, approximately 20,4196 novel hairpin sequences and 5441 novice mature products have been accompanied (6). As a member of a vast family of posttranscriptional modulators, miRNAs regulate various gene expressions at the posttranscriptional level by binding to the 3’ untranslated regions (UTR) of target messenger RNAs (mRNAs). miRNAs arrange diverse gene functions by mRNA digestion, inhibition of translation, or miRNA-mediated mRNA decay due to several factors, including complementary degree, the number and function of binding sites on target mRNA, which are positively correlated with each other (7,8). On the other hand, miRNAs can interact with the 5’ UTR of target mRNA, causing stimulation and activation of targeted proteins or inhibition of translation (9,10). Moreover, various miRNAs can interact with promoter protein structures, such as argonaute-2 and fragile X mental-retardation-related protein 1, which results in an indirect upregulation of the translation of the targeted genes (11).

To date, over 2500 miRNAs have been discovered and presented in the human body (12). Also, many novel computational programs have been found to predict target mRNAs of each specific miRNA, such as TargetScan (http://www.targetscan.org), miRanda (http:// www.microRNA.org), and TarBase (http://www.microrna.gr/tarbase). Since miRNAs have very complex regulatory mechanisms based on complementary base pairing between miRNA and target mRNA, an individual miRNA can arrange the expression and functions of multiple genes; and conversely, single genes can be regulated by many miRNAs (13). A diverse role of dysregulated miRNAs in several diseases in the human body was reported in a previous study (1,13,14). This review will present detailed information about miRNAs regarding structure and biogenesis, stages of synthesis and functions, expression profiles in the serum/plasma of living organisms, the diagnostic and prognostic potential as a novel biomarker, and therapeutic applications in various diseases.

## miRNA biogenesis and functions

**Figure 1:**
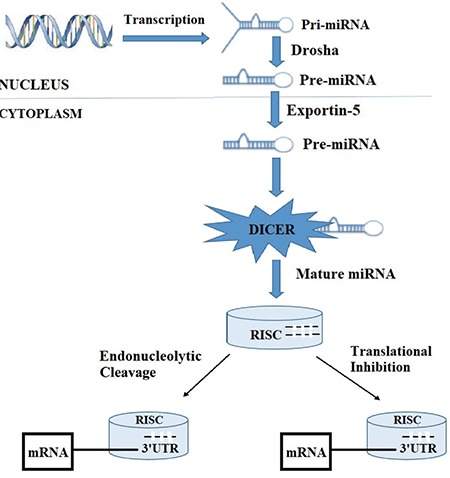
Biogenesis of miRNAs. UTR: untranslated regions, RISC: RNA-induced silencing complex

The biogenesis of miRNAs consists of multistep processes, including transcription, processing, splicing, export to the cytoplasm, maturation, and target binding (Figure 1). The first step is the synthesis of pri-miRNA, which is a large structure composed of several sequences for many miRNAs, from either an independent miRNA gene or parts of introns of protein coding for RNA polymerase II transcripts via the RNA polymerase II enzyme in the nucleus. The second step is the cleavage of pri-miRNA Drosha (RNA-specific RNase-III-type endonuclease) and its cofactor DGCR8 to produce the pre-miRNA that consists of nearly 70 nucleotides. Then, the pre-miRNA is transported to the cytoplasm and passes through nuclear pores in the membrane via exportin-5 and RanGTP binding protein (15). After that, the pre-miRNA undergoes further cleavage by Dicer (the cytoplasmic RNase-III endonuclease) into a double-stranded miRNA duplex (22 nucleotides) consisting of an active and functional mature guide strand and an inactive passenger strand in the cytoplasm. Subsequently, the binding of this duplex to the target, it is unwound, and the mature strand is loaded into the RNA-induced silencing complex. RNA-induced silencing complex is composed of Argonaute proteins and other modulatory effectors that organize the suppressor effect of this complex. Last, RNA-induced silencing complex guides the active strand to bind to its target mRNA through a complementary interaction between the miRNA and target mRNA seeding sequences. Ultimately, miRNAs show their effects on the human genome (16,17).

After synthesis and processing, extracellular miRNAs are secreted into the blood circulation by packaging them in different carriers, such as exosomes, microparticles, lipid vesicles, and bound with argonaute-2 or nucleophosmin-1 proteins, or with high- or low-density lipoproteins to protect them from digestion (18,19,20). Hence, miRNAs can maintain their stability in the blood under harsh conditions, such as extreme environmental basic pH, high temperature, multiple repeated freeze-thaw cycles, prolonged storage, and degradation by ribonuclease enzyme activity. However, exogenous free synthetic miRNAs are degraded easily by ribonucleases in the bloodstream (18,19,20). Most miRNAs are also produced from blood cells and tissues, such as the liver, heart, kidneys, and lungs (21).

miRNAs have important roles in various cellular events, such as embryogenesis, proliferation, vasculogenesis, apoptosis, cell growth and differentiation, and tumorigenesis. They also have potential roles in the cardiovascular system, such as angiogenesis, cardiac cell contractility, control of lipid metabolism, plaque formation, the arrangement of cardiac rhythm, and cardiac cell growth (22).

## miRNA and cardiovascular system development

A crucial role of many miRNAs in the development and function of heart and blood vessels in the human body was demonstrated in previous sequence-, microarray-, and other array-based profiling studies. miR-1 and miR-133, which have the highest expression levels in the heart, have opposite effects on cardiac cells; they promote and inhibit cardiac cell proliferation and differentiation, respectively (23). Moreover, several miRNAs, such as miR-1, miR-195, miR-133, miR-126, miR-16, miR-590, miR-199, miR-143, miR-208a, miR-499, miR-27-b, miR-497, miR-126, miR-30-d, miR-208b, miR-15a/b, and miR-16-1/2, partake in the regulation of cardiovascular system development, cardiogenesis, cardiac cell proliferation and differentiation, cell growth and integrity, cardiac cell communication, premature cardiac cell cycle arrest, and cardiac cell injury due to ischemia and/or hypoxia (24). Some miRNAs, such as miR-130, miR-145/miR-143 cluster, miR-210, and let-7f, play important roles in vascular smooth muscle cell proliferation and differentiation, migration, vessel development, and tubulogenesis (24,25).

## miRNA and cardiac hypertrophy and remodeling

Cardiac remodeling is very important for adaptive responses of the heart to stressful conditions or stressors. However, it leads to pathological hypertrophy, fibrosis, myocardial infarction, arrhythmias, tissue necrosis, cardiomyopathies, and heart failure if it continues for a long time. Cardiac remodeling is divided into cardiomyocyte apoptosis, hypertrophy, interstitial fibrosis, and regeneration. Previous studies reported some involvement and role of various miRNAs in the physiological and pathological development of cardiac hypertrophy. While miR-26, miR-9, miR-208 a and b, miR-133 a and b, miR-1, miR-195, miR-199, miR-18b, miR-124, miR-223, miR-98, and miR-499 were found to regulate cardiac hypertrophy in the physiological or pathological setting, miR-24, miR miR-101a and b, miR-30, miR-133, miR-21 and miR-29 were reported to be involved in cardiac fibrosis (26). Moreover, miR-221, miR-590, miR-33-b, miR-320, miR-34a could participate in cardiac cell regeneration (27).

## miRNA and vascular disease

In the human body, endothelial cells and vascular smooth muscle cells play a crucial role in maintaining vascular cell survival, integrity, and functions. Abnormal miRNAs expression may lead to different CVDs, such as atherosclerosis, vascular inflammation, diabetes with vascular complications, and coronary and peripheral artery diseases. miR-200, miR-34a, miR-217, and miR-146a were reported as being highly expressed in the setting of endothelial cell senescence, which is characterized as uncontrolled apoptosis, severe inflammation, and reduced endothelial nitric oxide synthesis and release leading to endothelial dysfunction, atherosclerosis, and its complications (26).

Angiogenesis is affected by distorted endothelial cell dysfunction that may cause increased inflammation. This increased inflammation ultimately results in vascular disease, ischemic cardiac events, and atherosclerosis. miR-126, miR-132, miR-16, miR-130, miR-101, miR-424, miR-221-222, miR-17-92, miR-200-b, and miR-23-24 were specifically expressed in vascular cells/tissues that participate in angiogenesis (26). Furthermore, vascular inflammation is defined as the activation and infiltration of leukocytes, and the production and secretion of inflammatory cytokines, growth factors, and adhesive molecules, which may lead to changes in the expression of several miRNAs and their functions in endothelial cells. Previous studies found that miR-31, miR-21, miR-10a, miR-17, miR-424, miR-181b, miR-106a, miR-17-92, miR-146, miR-17-5b, miR-150, miR-155 played a role in the setting of vascular inflammation (26).

## miRNA and atherosclerosis

Atherosclerosis is a complex disease that has many steps for its development, such as endothelial cell dysfunction, inflammatory cells infiltration and migration, impaired vascular cell integrity, vulnerable plaque formation, oxidized foam cell formation, lipid metabolism abnormalities, and vascular smooth muscle cell differentiation, which may lead to ischemic heart disease, hypertension, aortic aneurysm, heart valve disease, peripheral artery disease, and stroke. Some studies reported an important role of miRNAs in both humans and animals in the development of atherosclerosis. In endothelial dysfunction, miR-31, miR-181-b, miR-10a/b, miR-126, and miR-17-3p were demonstrated to participate in this process (28). Moreover, miR-122 and miR-33a/b were found to be important regulators of cholesterol homeostasis (28). Plaque development is one of the key steps in developing atherosclerosis and miR-26a, miR-221, miR-155, miR-21, and miR-125a-5p expression was altered in this setting (28). Moreover, miR-145, miR-127, miR-100, and miR-133a/b were reported as dysregulated miRNAs in plaque instability and rupture, which may result in acute coronary syndromes (28). In atherosclerosis, activated oxidized low-density lipoprotein containing foam cells can secrete different inflammatory cytokines, including those that participate in neoangiogenesis. Several various miRNAs, such as miR-210, miR-222, miR-155, miR-27a/b, and miR-221, may accompany foam cells participating in neoangiogenesis (28).

## miRNA and hypertension

Hypertension is one of the most important risk factors for developing atherosclerotic heart disease, heart failure, stroke, and peripheral artery disease. Arterial stiffness, aging, inflammation, the renin-angiotensin-aldosterone system, and endothelial dysfunction accompany the pathogenesis of hypertension. In recent years, the altered expression of some miRNAs, such as miR-132, miR-155, miR-212, miR-143/145, and miR-145, have been reported as regulators of blood pressure in humans by using the renin-angiotensin-aldosterone system (29). Furthermore, a previous study described miR-145 as a mediator of hypertension-induced vascular impairment (30). A relation of single nucleotide polymorphisms in miRNAs binding sites and essential hypertension was also demonstrated in the literature. Also, these single nucleotide polymorphisms located in miRNAs binding sites can frequently lead to changes in the blood pressure level (31). In the serum or plasma of humans, some miRNAs such as miR-130a, miR-210, miR-150, miR-191, miR-23b, miR-1246, and miR-451, were detected as highly expressed and defined as biomarkers for the early and precise diagnosis of hypertension (26). Furthermore, miRNAs were found to be strong potential non-invasive biomarkers for the early and correct detection of stroke related to hypertension (32). In a recent study, miR-30a, let-7b, and miR-126 were reported as useful biomarkers for hypertension-related ischemic stroke (33).

## miRNA and congenital heart disease

Congenital heart disease (CHD) consists mainly of congenital malformations, which have a prevalence rate of eight in every 1000 children (34). CHD is also related to both raised maternal and child mortality and morbidity in the world (34). The most common subtypes of CHD are atrial septal defects, ventricular septal defects, patent ductus arteriosus, tetralogy of Fallot, coarctation of the aorta, pulmonary valve atresia, and transposition of the great arteries. An association between altered expression of the miRNAs and ventricular septal defects was reported in some studies (35). miR-1-1, miR-181c, miR-19-b, miR-29-c, miR-195, miR-498, miR-133a, miR-92, miR-1-2, miR-133a-1/miR-1-2, miR-133a-2/miR-1-1, miR-1-1/miR-181c, miR-155-5p, miR-379-5p, miR-222-3p, miR-433, miR-409-3p, miR-487b, and miR-17-92 members were found dysregulated in the setting of ventricular septal defects. Moreover, NOTCH1, GATA3, ZFPM2, and HAND1 were present as the targets of miRNAs in the development of ventricular septal defects (34,35). For atrial septal defect, hsa-let-7a, hsa-let-7b, and miR-486 were found to be upregulated in the human body (36).

Down syndrome, or trisomy 21, which is one of the most important syndromes, including congenital heart defects, is characterized by an extra copy of chromosome 21. It has a variety of symptoms and clinical features and is diagnosed using specific techniques, such as fluorescence in situ hybridization. The rate of CHD in the setting of Down syndrome is between 40% and 60% (36). A previous study demonstrated the increased expression of specific miRNAs, such as let-7c, miR-155, miR-802, miR-99a, and miR-125b-2, in Down syndrome (37).

Tetralogy of Fallot, which is composed of large and non-restrictive ventricular septal defects, right ventricular outflow tract obstruction, an overriding aorta, and right ventricular hypertrophy, is the most commonly seen cyanotic CHD. It comprises approximately 5% to 7% of CHD (38). O'Brien et al. (39) reported an important link between miRNAs and tetralogy of Fallot. They detected 61 miRNAs that have important expression level changes in children with tetralogy of Fallot when compared with those without the disease. Furthermore, 33 miRNAs were reported significantly downregulated in myocardial tissue samples in patients with tetralogy of Fallot when compared with a healthy myocardium (39). While miR-22, miR-138, miR-222, miR-375, miR-421, and miR-424/424* were demonstrated to be upregulated, miR-1, miR-206, and miR-940 were downregulated under this condition (39,40).

Bicuspid aortic valve is another commonly seen CHD with a prevalence between 1% and 2% in the general population. The main complications of this disease are aortic valve diseases, such as calcific stenosis or insufficiency and thoracic aortic aneurysms (41). Novel dysregulated miRNAs, eight miRNAs were upregulated, and 27 miRNAs were downregulated (miR-141 was the most common), were detected in bicuspid aortic valve leaflets of the aortic valve in a recent study (42). An association between miRNAs and the bicuspid aortic valve was assessed in a study by Nigam et al. In this study, miR-26a, miR-30b, and miR-195 levels were importantly decreased in the aortic valve leaflet of these patients (43). In their study, Zhu et al. (44) suggested a potential use for miRNAs as biomarkers to identify fetal CHD during the prenatal period from maternal serum. They found that miR-19b, miR-22, miR-29c, and miR-375 were importantly upregulated in mothers carrying fetuses with CHD. This study was significant concerning the promising possibility of using miRNAs in clinical practice to identify CHD during the prenatal period (44). Finally, altered miRNA expression (up-, down-, or coexpression) has a common and specific effect on cell communication pathways via signals during the development of the CHD.

## miRNA and pulmonary hypertension

Pulmonary arterial hypertension is one of the leading causes of death worldwide. It is divided into several subtypes, including idiopathic pulmonary arterial hypertension, heritable pulmonary arterial hypertension, and pulmonary arterial hypertension associated with other diseases (45). Although genetic, environmental, and epigenetic factors were supposed to be etiopathologic causes of this disease, certain factors were not found (46). Inflammation, endothelial cell proliferation of the pulmonary artery, proliferation, severe constriction, contraction, and migration of pulmonary vascular smooth muscle cells and fibroblast activation, migration, and proliferation are well known pathogenetic mechanisms of pulmonary arterial hypertension (46). A potential pathogenetic role of miRNAs has been investigated and reported in previous studies. miR-21, miR-204, miR-17-92, miR-145, miR-124, and miR-210 were shown to be dysregulated miRNAs in the proliferation, migration, and contraction of pulmonary artery smooth muscle cells (46). Moreover, miR-503, miR-27a, miR-424, miR-17-92, and miR-21 were expressed in endothelial cells and participated in proliferation and resistance to apoptosis (46). In recent studies, the early diagnostic and prognostic value of miRNAs as novel biomarkers in pulmonary arterial hypertension has been reported (47). The study by Rhodes et al. (47) demonstrated an important correlation between the decreased expression level of miR-150 and poor prognosis in patients with pulmonary arterial hypertension. In addition, Schlosser et al. (48) reported a positive relation of miR-26a with elevated right ventricular systolic pressure, right ventricular hypertrophy, and exercise capacity using the six-minute walk distance. miRNAs can play a key role not only in the correct and early diagnosis and risk stratification but also in the treatment of pulmonary arterial hypertension. They can be novel therapeutic drugs used as agonists and antagonists in humans based on their capability of affecting several genes within a genome, which makes them more beneficial.

## miRNA and acute myocardial infarction

Acute myocardial infarction, which is one of the results of atherosclerosis, is a life-threatening disease with high mortality and morbidity. Vulnerable atherosclerotic plaque rupture, acute coronary artery occlusion due to the formation of a thrombus, severe coronary vasoconstriction, and an imbalance in the supply and demand are important pathological mechanisms of acute myocardial infarction. Cardiac remodeling, including heart chamber dilatations and ventricular wall thinning due to severe necrosis and fibrosis following acute myocardial infarction, can lead to systolic heart failure. Early and precise diagnosis and timely and appropriate treatment are very crucial to prevent complications of acute myocardial infarction, including death. In recent years, many biomarkers have been used to predict mortality and morbidity rates in the world. As novel biomarkers, miRNAs have been investigated in the setting of acute myocardial infarction in previous studies. Altered expression of miRNAs (miR-499, miR-636, miR-380, miR-133a, miR-17, miR-21, miR-29b, miR-192, miR-194, miR-499, miR-1915, miR-34a, miR-423, miR-328, miR-134, miR-1254, miR-1, miR-181c, miR-208b, miR-566, miR-7-1, miR-92a, miR-455-3p, miR-126, miR-423-5p, miR-636, miR-486, and miR-1291 were upregulated, whereas miR-197, miR-106, and miR-223 were downregulated) have been detected in serum/plasma of patients with acute myocardial infarction and have been used as new biomarkers for predicting major adverse cardiovascular events in past studies (26). Clinical studies, including miRNAs that had both diagnostic and prognostic significance in the setting of acute myocardial infarction, are shown in Table 1 (49,50,51,52,53,54,55,56). Coskunpinar et al. (57) reported an increase of miR-221-3p in subjects with acute myocardial infarction, which were correlated with ejection fraction (inversely), troponin, and risk scores. In addition, it was reported to be a good candidate as a biomarker in this setting (57). The study by Townley-Tilson et al. (58) demonstrated a good correlation of miR-1 and miR-133, which play key roles in cardiac muscle growth and differentiation, with myocardial infarct size. They reported that left ventricular ejection fraction and mortality were related in their study (58). Moreover, a potential role of miR-21 as a novel predictive biomarker for cardiac remodeling following acute myocardial infarction was shown in a study by Liu et al. (59). Similar to the previous study, miR-21 was positively correlated with troponin and had strong diagnostic accuracy (59). Widera et al. (60) reported an association between an increased level of miR-208 and major adverse cardiovascular events at six months, including mortality or heart failure. Furthermore, an elevated miR-499 was found to be a more reliable biomarker when compared with traditional ones in acute myocardial infarction, which had both higher sensitivity and specificity than troponin for the early diagnosis of acute myocardial infarction (49). miR-499 was also presented as a new robust biomarker for detecting perioperative MI, especially in cardiovascular surgery (49). Some studies were performed to identify the differences in the expression of miRNAs between non-ST segment elevation (NSTEMI) and ST segment elevation MI (STEMI). miR-133a, miR-208b, miR-499, miR-451, and miR-134 levels were found to be higher in STEMI compared with NSTEMI. However, miR-145 was found to be lower in the STEMI group than those in the NSTEMI group (61).

miRNAs can play an important role not only in the diagnosis but also in the prognosis of acute myocardial infarction. miR-208b was reported to be a biomarker for predicting mortality after other risk adjustments in a past study (51). Moreover, miR-133a was significantly demonstrated to be associated with all-cause mortality following age and gender adjustments (62). However, in contrast to these studies, other studies did not support the findings of previous studies about the potential prognostic role of miR-133a and miR-208b (62,63). According to the results of many studies, miRNAs could be beneficial for predicting short- or middle-term prognosis regarding mortality in acute myocardial infarction.

The possible mechanisms of miRNAs for use as biomarkers for acute myocardial infarction include delivering circulating miRNAs to recipient target cells to arrange the translation of proteins and having specific secretory pathways or passive secretion of miRNAs into blood circulation immediately after cell disruption and death (25). Cyclic changes in the expression of miRNAs associated with myocardial viability and growth, fibrosis, and remodeling can affect ventricular performance and the contraction ability of the heart (64).

## miRNAs and arrhythmias

Arrhythmia is an abnormal heart rhythm that may lead to several complications, including sudden death. It may be classified as tachycardia, bradycardia, supraventricular, or ventricular, according to electrocardiographic and/or electrophysiologic findings. A relation of dysregulated miRNAs with the development of arrhythmia was reported in the literature (65). While miR-133, miR-31, miR-483, miR-208b, and miR-328 were upregulated in the setting of ventricular tachycardia, postoperative atrial fibrillation and atrial fibrillation, respectively, and miR-150, miR-23a, and miR-1 were downregulated in atrial fibrillation and supraventricular tachycardia orderly. Furthermore, miR-1, miR-27 and 28, miR-34, miR-146, miR-206, miR-590, and miR-155 were upregulated, whereas miR-1, miR-26, miR-30, miR-106, miR-29, miR-125 and 126, miR-133, miR-199, miR-590, and miR-409 were downregulated in atrial fibrillation (65).

Atrial fibrillation, which is a chronic and the most common form of arrhythmia, has many complications, such as stroke, heart failure, acute myocardial infarction, coronary artery diseases (CAD), and death. Several factors, such as anatomical and electrical remodeling, structural changes, autonomic nervous system dysfunction, calcium handling impairment, severe inflammation, and variations at the level of nucleotides in miRNA and its targeted genes, are related to the initiation and progression of atrial fibrillation (65). miRNAs have significant roles in both the development and progression of the disease. miR-29 was demonstrated to be a stimulator of atrial fibrillation by arranging apoptotic and fibrotic cardiac genes (66). In addition to miR-483, miR-23a and miR-26a can be modulators of postoperative atrial fibrillation (67). miRNAs were supposed to be regulators of atrial remodeling by affecting Ca+2 channel protein expression. miR-328, miR-30d, and miR-499 play crucial roles in this pathway (46). Moreover, miR-21, which was reported to have elevated levels in both human and animal studies, may contribute to structural remodeling, including fibroblast activation and proliferation, interstitial fibrosis, and cardiac hypertrophy of the atrial myocardium by using extracellular signal-regulated or mitogen-activated protein kinase pathways resulting in atrial fibrillation (68). A decreased level of miR-150 in platelets of patients with atrial fibrillation was also shown in a previous study, which was thought to be associated with inflammation, fibrosis, and increased platelet function (69).

## miRNAs and aortic aneurysm

Artery aneurysms are organized as segmental or diffuse symmetrical dilatations of the arterial wall. They are most commonly seen in the infra-renal part of the abdominal aorta. The precise pathological mechanism of this disease is severe intraluminal pressure exceeding the expandable capacity of the arterial wall, which results in weakness of the vessel structure. Abdominal aortic aneurysms are one of the most common causes of death, especially in men over 65 years old. Although it can be asymptomatic over a long time, its acute clinical manifestations, such as acute aortic rupture or dissection, may lead to death without acute treatment. thoracic aortic aneurysm is also a silent, commonly seen, and curative aortic disease. It can be subdivided into portions based on the affected segment of the thoracic aorta (70). Aneurysms are seen more common in the abdominal aorta compared with the thoracic portion. Atherosclerosis is the main pathogenetic mechanism in both the development and progression of abdominal aortic aneurysms. Traditional common risk factors of aortic aneurysms are similar to CADs such as age, gender, genetics, hypertension, hyperlipidemia, smoking, and diabetes except for the ascending thoracic aorta, which is affected by genetic factors, connective tissue disease, hypertension, and the bicuspid structure of the aortic valve. Recent studies demonstrated that miRNAs made beneficial contributions to enable the early diagnosis and middle- to long-term prognosis and preventive potential of aortic aneurysms (70). While miR-205, miR-195, miR-221-222, miR-21, and miR-29 were found to be upregulated, mir-26a and miR-143/145 were downregulated in aortic aneurysms (70). miRNAs can participate in this process by affecting proliferation, apoptosis, differentiation, and functions of vascular smooth muscle cells. In addition, in thoracic aortic aneurysm, miR-491-3p, miR-338-5p, miR-433, miR-183, miR-553, and miR-30c had increased expression. On the other hand, miR-24, miR-143, miR-22, miR-93, and miR-145 were downregulated in this setting (71).

## microRNA and valvular heart disease

Valvular heart disease is one of the leading causes of cardiovascular mortality. Aortic and mitral valve disorders are seen more frequently than are those of right-sided valves. Calcific aortic valve stenosis is one of the most commonly seen valvular diseases in developed countries. Its risk factors are similar to those of CAD, and its treatment is possible through valve replacement using interventional and surgical techniques. Aortic valve stenosis is defined as the chronic, progressive narrowing of the valve orifice. Endothelial dysfunction, atherosclerosis, inflammation, oxidized lipid deposits, and impairment of calcium metabolism are the main pathophysiologic mechanisms of the disease (72). Moreover, excessive collagen synthesis, severe calcification due to apoptosis, osteogenic transdifferentiation of the valvular interstitial cells lead to valve remodeling, including extracellular matrix rearrangement and fibrosis (72). Finally, due to chronic constant inflammation and calcification, the mobility of leaflets decreases, valve stenosis occurs, and blood flow through the orifice decreases. Several dysregulated miRNAs, such as miR-22, miR-486, miR-210, miR-125, and miR-21, were present in recent studies (73). Moreover, miR-210, miR-21, and miR-133a, which play a key role in left ventricular remodeling and fibrosis, were reported as strong prognostic biomarkers in patients with aortic stenosis in the literature (72,73,74,75).

Mitral valve disease, which is classified as stenosis and insufficiency, is the other commonly seen valvular pathology. It can lead to moderate-advanced heart failure, arrhythmias, and sudden cardiac death. In mitral insufficiency, valve leaflet structure impairment or secondary pathologies due to left ventricle dilatation can be etiological factors for development. Systolic and diastolic heart failure, electrical abnormalities, and arrhythmias are the main clinical outcomes of mitral insufficiency. Mitral stenosis is another mitral valve disease, which has similar risk factors and pathophysiologic mechanisms to aortic stenosis. Changes in the expression levels of miRNAs in mitral valve leaflets in the setting of rheumatic mitral stenosis and mitral valve prolapse were reported in some studies (73). While miR-26a-5p, miR-133b, miR-145-5p, miR-30c-5p, miR-1, miR-23b-3p, and miR-125-5 were downregulated, miR-4484, miR-3613-3p, and miR-466 were upregulated in both the left and right atrial appendage tissue of patients with rheumatic mitral valve disease indicating surgery in previous studies (73,76). Furthermore, some miRNAs, such as miR-34c-3p, miR-656, miR-500, miR-3174, miR-379-3p, and miR-664a-3p, were found to be highly expressed in patients with myxomatous mitral valve leaflet prolapse (77). In contrast, miR-1193, miR-646, miR-203, miR-939, miR-4298, miR-17, miR-505, and miR-1273e were downregulated in the setting of mitral valve prolapse (78).

## miRNA and restenosis

Vascular remodeling and restenosis are pathophysiologic results of atherosclerosis. Although the use of drug-eluting coronary stents during the percutaneous coronary intervention can decrease its probability, the complication rate of restenosis remains high. Severe neointimal proliferation and hyperplasia, vascular remodeling, increased vascular smooth muscle cell proliferation and migration, and chronic inflammation after the coronary stent implantation procedure are the main causes of restenosis. Clinical results of restenosis may include recurrent ischemia and angina, the need to repeat revascularization, and acute coronary syndrome. A predictive role of circulating miR-143 level for in-stent restenosis in coronary or peripheral artery diseases was reported in a previous study (79). Moreover, an important role of miR-145, miR-221, miR-663, miR-599, miR-143, miR-15b, miR-9, miR-206, miR-181b, miR-16, miR-31, miR-146a, miR-222, and miR-22 in the differentiation of vascular smooth muscle cell was demonstrated in a previous study (79). In addition, miR-133 was found to be a dysregulated miRNA in the setting of restenosis (79). This study demonstrated that an elevated transcoronary miR-133 level was a good predictor of the need for target lesion revascularization due to in-stent restenosis. Moreover, miR-23b, miR-125a-5p, miR-195, and miR-663 were shown to be dysregulated in the setting of restenosis (79).

miR-126, miR-17/92a, miR-221 and 222, miR-16, and miR-21 were dysregulated miRNAs expressed in endothelial cells (79). Experimental studies reported that miR-21 had a stimulator role in vascular smooth muscle cell proliferation using specific pathways (80). The use of antisense oligonucleotides against miR-21 to target the damaged area was found to be beneficial for inhibiting vascular remodeling, vascular smooth muscle cell proliferation, and neointimal hyperplasia in-stent restenosis in animal models (80). In addition, Wang et al. reported beneficial inhibitor effects of locally delivered anti-miR-21 as a coated stent in the stenotic artery, which decreased in-stent restenosis with re-endothelialization (81). Also, the study by He et al. (82) showed an increased level of miR-21 in patients with in-stent restenosis. Endothelial recovery is one of the protective mechanisms for preventing restenosis. The diagnostic potential of miR-21 for in-stent restenosis was also reported in a previous study (80). Elevated peripheral miR-92 and miR-195 levels were found to be good independent predictors for two years of target vessel revascularization (83). Similar to this finding, increased transcoronary expression of miR-133a had both diagnostic and prognostic significance for target vessel revascularization (84). Decreased levels of miR-145 and miR-143 in the coronary artery were also concordant with previous findings regarding both the diagnosis and prognosis (82). Some miRNAs, such as miR-16, miR-21, miR-92a, and miR-221 and 222, were found to play key roles in this process. Moreover, miR-21, miR-92a, miR-16, and miR-126 can participate in angiogenesis, which is an important part of restenosis (79).

## miRNA and heart failure

heart failure is defined as a clinical syndrome and is characterized by the heart, not providing adequate blood, oxygen, and nutrients to meet the metabolic requirements of the human body. It is divided into three subdivisions: systolic, diastolic, and mid-range ejection fraction heart failure. Heart failure is one of the most common causes of death in the world. CAD, hypertension, valvular heart disease, arrhythmias, viral infections such as myocarditis, cardiomyopathies, and some cardiotoxic drugs or materials are well known etiologic factors of heart failure (85). A significant independent role of circulating miRNAs for both the diagnosis and prognosis of heart failure was reported in recent studies (86,87,88,89,90,91,92,93,94,95,96,97,98). The results of these studies also provided strong evidence about the key role of miRNAs in the development and progression of the disease. In addition, a relation between miRNAs and clinical, imaging, and laboratory findings was demonstrated in previous studies (86,87,88,89,90,91,92,93,94,95,96,97,98). While miR-18a, miR-26b, miR-106a, miR-30e, miR-27a, miR-199a, and miR-652 were found to be decreased, miR-30d, miR-126, miR-1254, miR-37, miR-30c, miR-223-3p, miR-301a-3p, miR-210, miR-145-5p, miR-29a-3p, miR-1306-5p, miR-26b-5p, miR-199a-3p, miR-92a-3p, miR-146a, and miR-221 were upregulated in patients with heart failure (86,87,88,89,90,91,92,93,94,95,96,97,98). A summary of clinical studies that showed miRNAs having both diagnostic and prognostic significance in patients with heart failure is shown in Table 2 (86,87,88,89,90,91,92,93,94,95,96,97,98). After 48 hours from admission for heart failure, declining in miR-18a, miR-652, miR-18b, miR-301a, let-7i, miR-223, and miR-423 levels were found to be significant independent biomarkers of six months of mortality (91). miR-650, miR-1228, miR-662, miR-518, miR-3148, miR-21, and miR-299-3p were reported to be correlated with N-terminal pro-brain natriuretic peptide (NT-pro-BNP) in the study by Cakmak et al. (97). Also, miR-182 was found to be an important prognostic marker for six months cardiovascular mortality in the same study (97). In addition to the diagnostic and prognostic features of miRNAs in heart failure, a relation between miRNAs and echocardiographic left ventricular anatomic structural change in systolic heart failure was investigated (99). miR-182, miR-568, and miR-200a were negatively correlated with left ventricular mass index, whereas miR-155 and miR-595 were found to be positively correlated with left ventricular mass index (99). In other studies, altered expression levels (up- or downregulated) of miR-423, miR-1254, and miR-1306 were reported as prognostic markers in patients with heart failure (1,98).

The diagnostic performance of NT-pro-BNP for heart failure was enhanced by combining it with miRNAs, such as miR-146a, miR-221, miR-375, miR-423-5p, miR-30c, and miR-328 as presented in past studies (1,89). Moreover, levels of circulating miRNAs can change with some medications. Left ventricular assist devices can affect miRNAs levels (miR-499, miR-208a and b, miR-133, and miR-1), either muscle-specific or non-specific (100). A reverse remodeling after cardiac resynchronization treatment, which was defined as “responders,” was reported to be related to changes in miRNAs levels (101). In this study, miR-92a, miR-29a, miR- 26b, miR-145, and miR-30e levels rose in the responder group when compared with non-responders (101). Also, initial levels of miR-30d and miR-1306 were demonstrated to be associated with the reverse left ventricular remodeling, which was defined as “responder,” in end-stage chronic heart failure patients. They also had a prognostic role for one-year all-cause mortality in patients with acute heart failure (101).

## miRNAs and therapeutic potential

miRNAs are promising, novel therapeutic agents, which can affect multiple genes by using different signaling pathways (87). Treatment options with miRNA drugs can be classified into two groups: miRNA depression to decrease the levels of miRNAs upregulated in cardiac diseases, and replacement of missing miRNA to restore the expression of miRNAs reduced in disease condition (87). Nowadays, miRNAs have been used not only for diagnostic and prognostic purposes but also for therapeutic aims in various CVD. Many techniques, such as viral vectors, vesicles, antagomirs or mimics, plasmids and sponges created to be used as vehicles for miRNAs to specific target tissues or organs without digestion, which increase bioavailability and bioefficacy (102). miRNAs therapeutic modulation techniques were used in the settings of atherosclerosis, acute myocardial infarction, restenosis, vascular remodeling, arrhythmias, hypertrophy and fibrosis, angiogenesis and cardiogenesis, aortic aneurysm, pulmonary hypertension, and ischemic injury (102,103,104,105,106,107,108,109,110,111,112,113,114,115,116,117,118,119,120,121,122,123,124,125,126,127) (Table 3).

Moreover, miRNAs can provide improved clinical outcomes. On the other hand, as a delivery system, the off-target impact and inductor effect of the immune system are big problems for miRNA-based drugs for use in clinical practice (103). While permanent impact on target tissue or organ system, easy to be regulated, ability to affect multiple pathways to show their effect, strong impact with relatively lower doses are the main positive features of miRNAs, having an off-target effect, risk of toxicity with the delivery agent or miRNAs themselves, difficult detection of tissue-specific pharmacodynamic effect, and harsh delivery of miRNAs to target tissues are challenges of these therapeutic systems (88).

## miRNAs and future perspectives

Circulating miRNAs are promising novel biomarkers for purposes of both diagnosis and prognosis of CVD. Cell or tissue specificity, stability in serum or plasma, resistance to degradative factors, such as freeze-thaw cycles or enzymes in the blood, and fast-release kinetics, provide miRNAs with the potential to be surrogate markers for both the early and accurate diagnosis of disease and prediction of middle- or long-term prognosis. Moreover, combining miRNAs with traditional biomarkers can be the logical approach to improve risk stratification and long-term prognosis. miRNA-based therapeutics may be more beneficial to treat CVD using novel platforms and computational tools in combination with traditional methods of analysis.

## Figures and Tables

**Table 1:**
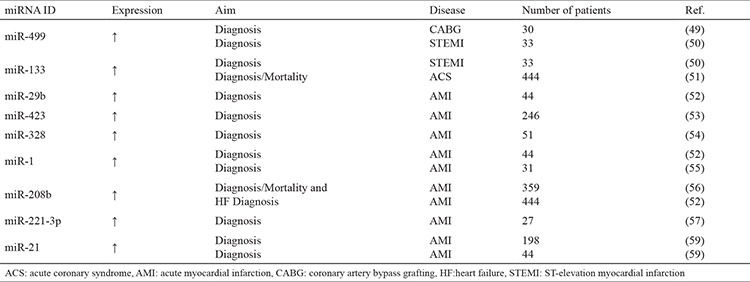
Diagnostic and prognostic significance of miRNAs in acute myocardial infarction

**Table 2:**
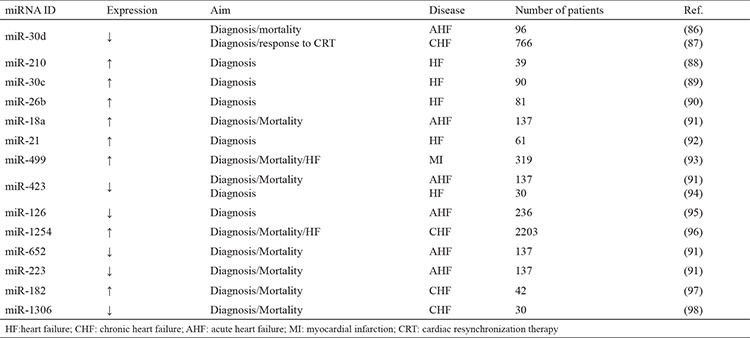
Diagnostic and prognostic significance of miRNAs in heart failure

**Table 3:**
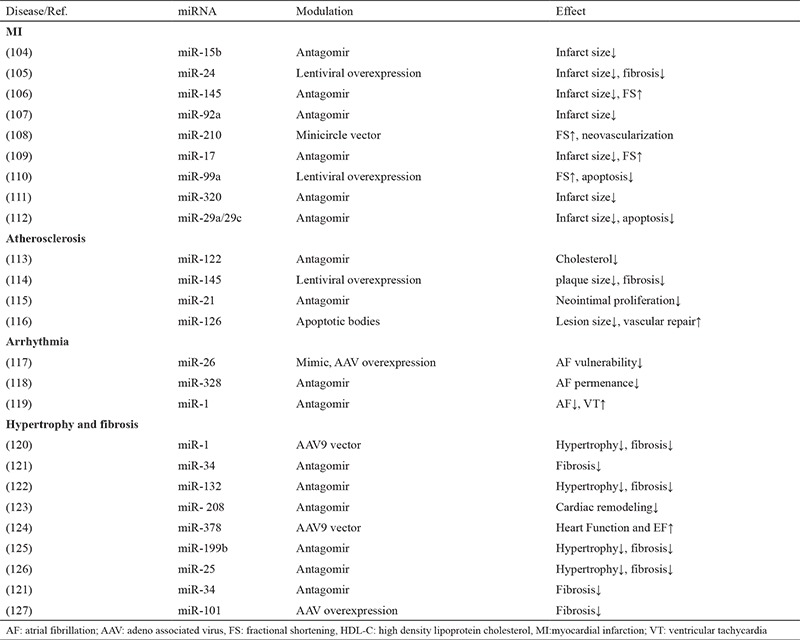
Therapeutic applications of miRNAs in cardiovascular diseases
